# Rhesus monkeys as a translational model for late‐onset Alzheimer's disease

**DOI:** 10.1111/acel.13374

**Published:** 2021-05-05

**Authors:** Dylan C. Souder, Isabelle A. Dreischmeier, Alex B. Smith, Samantha Wright, Stephen A. Martin, Md Abdul Kader Sagar, Kevin W. Eliceiri, Shahriar M. Salamat, Barbara B. Bendlin, Ricki J. Colman, T. Mark Beasley, Rozalyn M. Anderson

**Affiliations:** ^1^ Division of Geriatrics Department of Medicine SMPH Madison WI USA; ^2^ Biology of Aging Laboratory Center for American Indian and Rural Health Equity Montana State University Bozeman MT USA; ^3^ Department of Biomedical Engineering University of Wisconsin Madison Madison WI USA; ^4^ Department of Pathology Laboratory Medicine University of Wisconsin Madison Madison WI USA; ^5^ Neurological Surgery University of Wisconsin Madison Madison WI USA; ^6^ Wisconsin National Primate Research Center University of Wisconsin Madison Madison WI USA; ^7^ Department of Biostatistics University of Alabama Birmingham AL USA; ^8^ GRECC Birmingham/Atlanta Veterans Administration Hospital Birmingham AL USA; ^9^ GRECC William S. Middleton Memorial Veterans Hospital Madison WI USA

**Keywords:** aging, amyloid plaque, astrocyte, microglia, multiphoton imaging, nonhuman primate

## Abstract

Age is a major risk factor for late‐onset Alzheimer's disease (AD) but seldom features in laboratory models of the disease. Furthermore, heterogeneity in size and density of AD plaques observed in individuals are not recapitulated in transgenic mouse models, presenting an incomplete picture. We show that the amyloid plaque microenvironment is not equivalent between rodent and primate species, and that differences in the impact of AD pathology on local metabolism and inflammation might explain established differences in neurodegeneration and functional decline. Using brain tissue from transgenic APP/PSEN1 mice, rhesus monkeys with age‐related amyloid plaques, and human subjects with confirmed AD, we report altered energetics in the plaque microenvironment. Metabolic features included changes in mitochondrial distribution and enzymatic activity, and changes in redox cofactors NAD(P)H that were shared among species. A greater burden of lipofuscin was detected in the brains from monkeys and humans of advanced age compared to transgenic mice. Local inflammatory signatures indexed by astrogliosis and microglial activation were detected in each species; however, the inflamed zone was considerably larger for monkeys and humans. These data demonstrate the advantage of nonhuman primates in modeling the plaque microenvironment, and provide a new framework to investigate how AD pathology might contribute to functional loss.

## Graphical abstract

Souder et al. report the outcome of a cross‐species comparison of the local impact of amyloid plaques in the brains of APP/PSEN mouse model, aged monkeys, and human subjects diagnosed with Alzheimer's disease. Microenvironment mitochondrial clustering and altered redox status were common to each species, but lipofuscin accumulation and elevated inflammation were more pronounced in monkeys and humans, indicating that age contributes to amyloid pathology.

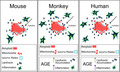

## 1 INTRODUCTION

Alzheimer's disease (AD) is a progressive neurodegenerative condition that accounts for most cases of dementia in the United States (Nichols et al., 2019). AD manifests as either early‐onset familial AD or as late‐onset AD that is more prevalent and accounts for approximately 95% of occurrences (Harman, 2006). Familial AD is the result of inherited mutations in the amyloid precursor protein (APP) and presenilins (PSEN1 and PSEN2) (Tanzi & Bertram, 2001); however, the cause of late‐onset AD remains elusive. Genetic risk factors including apolipoprotein (APOE4) ε4 and triggering receptor expressed on myeloid cells 2 (TREM2) have been found to substantially increase the risk of disease, but do not fully account for AD pathogenesis and progression (Karch & Goate, 2015). Environmental and lifestyle factors have also been linked to disease risk and cognitive decline (Brown et al., 2013). Obesity in particular has been shown to increase the risk and severity of dementia as well as impacting the age of onset (Whitmer et al., 2008). This diverse set of genetic and environmental risk factors presents a challenge for researchers modeling neurodegenerative conditions. The majority of animal models used in AD research are transgenic mice expressing mutant versions of APP, presenilins, and human tau proteins that drive early‐onset plaque and tangle formation (Duff, 2001); however, aged mice are seldom studied. These issues may explain in part why therapeutics developed using the transgenic mouse models have not performed as expected in clinical trials (Drummond & Wisniewski, 2017).

Aging is the strongest risk factor for non‐communicable disease, particularly AD, where the prevalence doubles nearly every five years after age sixty‐five years (Qiu et al., 2009). While several features of aging are similar between rodent and humans, important differences also exist. Perhaps, the most salient difference between rodent and human aging is the formation of amyloid plaques. Indeed, the amyloid beta region of mouse APP differs from human in three amino acids and, as a result, is unable to aggregate (Duyckaerts et al., 2008). Although amyloidosis is a common feature of aging in humans, it is not always associated with cognitive deficits, and many individuals with significant amyloid burden are cognitively normal (Jack et al., 2017). Importantly, most AD mouse studies involve early‐onset pathologies, excluding the possibility of discovering how aging impacts the emergence of AD pathology, and missing the contribution of the aging brain environment that is the backdrop for onset and progression of the most prevalent form of AD.

Nonhuman primates closely mirror the progression, cognitive decline, and neuropathology observed in human brain aging (Price et al., 1991). The rhesus monkey longitudinal aging cohort at the Wisconsin National Primate Research Center has been extensively investigated for signatures of aging and age‐related disease (Colman et al., 2009, 2014; Mattison et al., 2017). In terms of brain aging, monkeys from this cohort show age‐related brain atrophy, heavy metal deposits, and interactions between peripheral metabolism and brain aging (Bendlin et al., 2011; Kastman et al., 2012; Sridharan et al., 2012; Willette et al., 2012). Independent studies have shown that, similar to humans, monkeys display age‐related accumulation of amyloid plaques with immunoreactivity to ApoE (Poduri et al., 1994). We have previously shown that amyloid accumulation is associated with astrocyte activation in monkeys and that the local impact on inflammation is blunted by caloric restriction, an intervention that also delays brain atrophy (Colman et al., 2009; Sridharan et al., 2013). Work conducted elsewhere has shown that microinjection of fibrillary amyloid beta into the aged macaque cortex results in neuronal loss, tau phosphorylation, and microglial proliferation, displaying neurotoxicity that, in contrast, is not observed in young macaques (Geula et al., 1998). This appears to be a primate‐specific outcome as the same treatment in aged rats failed to produce the same results. The suitability of rhesus monkeys as a means to understand AD is further supported by a more recent study demonstrating that macaques develop Braak stage III/IV tau pathology (Paspalas et al., 2018). Together these data suggest that rhesus monkeys may capture key elements of human AD that are not reflected in genetically manipulated rodent models.

The purpose of the present study was to investigate species‐specific differences in the local impact of AD pathology. Specifically, we compared the microenvironment adjacent to neuritic plaques in the APP/PSEN1 mouse, aged rhesus monkeys, and in brain tissue from humans with diagnosed AD. In situ techniques to quantify metabolic and inflammatory indices, specifically astroglial and microglial activation, were employed to determine the extent to which murine and nonhuman models reflect human AD, and to establish if there may be advantages to using aged nonhuman primates in AD research.

## 2 RESULTS

This multi‐species study used brain tissues taken from APP/PSEN1 male mice (6 months of age; total *n* = 6), male and female rhesus monkeys (~31 years of age; total *n* = 5), and male and female human subjects (~86 years of age; total *n* = 10) (Table S1). As a frame of reference, average lifespan of C57BL/6 mice is ~28 months and the average lifespan for rhesus monkeys in captivity ~26 years. Mice were maintained on standard diet under standard housing conditions. Monkey tissue was obtained from advanced‐aged monkeys from the Wisconsin National Primate Research Center rhesus monkey longitudinal aging cohort (Colman et al., 2009, 2014). Human tissue was acquired from the Wisconsin Alzheimer's Disease Research Center (ADRC) brain bank that includes cases from the ADRC clinical core. For this study, specimens were from individuals with a postmortem diagnosis of AD following the National Institute of Aging and the Alzheimer Association criteria for neuropathological AD (Hyman et al., 2012). Quantitative histological, histochemical, and immunodetection techniques were used to identify plaques and investigate the localized impact of amyloid on metabolic and inflammatory parameters.

### 2.1 Mitochondrial clusters surround amyloid plaques

**FIGURE 1:**
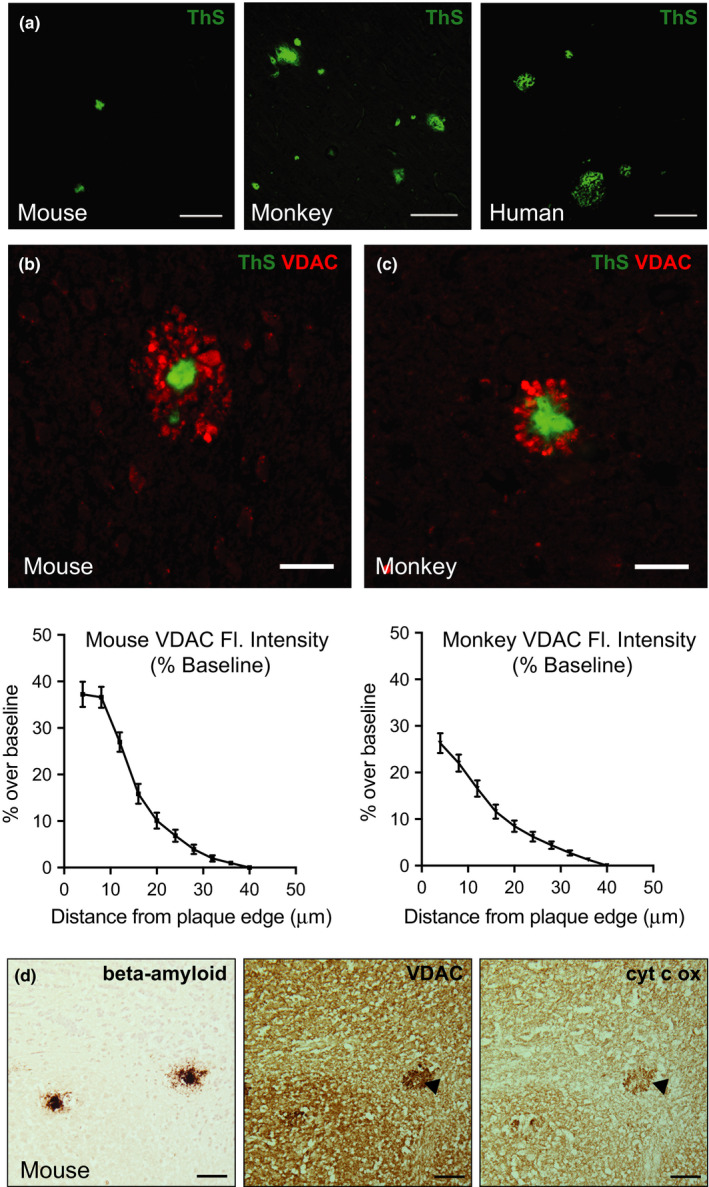
Mitochondria accumulate around amyloid plaques. (a) Thioflavin S (ThS) staining of dense‐core amyloid plaques in mice, monkey, and human AD cortex. Scale bar = 100 µm. (b) Immunofluorescent labeling of mitochondria (VDAC) and amyloid plaques (ThS) in cortical tissue of mice and (c) rhesus monkeys (40×) with quantification of fluorescent stain intensity relative to baseline. (d) Adjacent cryosections of mouse cortex immunostained for beta‐amyloid, VDAC, and cytochrome c oxidase enzyme activity. Black arrow indicates plaque‐associated mitochondria. Scale bar = 20 µm. Mouse (*n* = 5–6); monkey (*n* = 5); human (*n* = 10) biological replicates with 39–294 plaque ROIs quantified within species; data are shown as average ± SEM

Amyloid plaques were identified by fluorescent detection of thioflavin S (ThS) stain of tissue sections from mouse somatosensory cortex, monkey temporal cortex, and human temporal cortex (Table S1). Greater diversity in amyloid plaque morphology and size was observed in monkeys and humans relative to transgenic mice (Figure 1a). In cortical sections from APP/PSEN1 mice (39 individual plaques from 5 mice), the mean plaque size was 1211 ± 839 µm^2^ (mean ± *SD*). In cortical sections from rhesus monkeys, (69 individual plaques from 5 monkeys), amyloid plaque size was more variable than in mice, and mean plaque size was significantly greater at 1957 ± 1403 µm^2^. In humans, (294 individual plaques from 10 subjects), mean plaque size was 2111 ± 1239 µm^2^, similar in size and in variance to what was observed in monkeys (Figure S1). A growing literature supports the concept that mitochondrial dysfunction is an important feature of AD pathology (Swerdlow et al., 2014), although cross‐species studies have not explored this idea. In this study, mitochondrial distribution was assessed in rodent, monkey, and human specimens using antibodies against voltage‐dependent anion channel 1 (VDAC1), an abundant mitochondrial outer membrane transporter protein. Mitochondria (red) were detected throughout the tissue specimens, but also in high‐density ring clusters or crown‐like structures (Figure 1b,c). Co‐staining for amyloid plaques (green) confirmed that these mitochondrial crowns were completely surrounding amyloid plaques in both species. High autofluorescence in the brains from human subjects prevented quantitation of fluorescent detection of mitochondria (Figure S1); however, immunohistochemical staining for VDAC1 revealed a similar increase in mitochondria surrounding plaques mirroring the enrichment detected in mice and monkeys (Figure S1).

The VDAC stain detects the presence of mitochondria but does not indicate metabolic activity status of these organelles. Procedures involved in procurement of monkey and human cortical tissue prohibited enzyme activity‐based quantitation; however, the mouse tissues were flash‐frozen so it was possible to determine whether the plaque‐associated population of mitochondria was functional. Histochemical detection of enzymatic activity of complex IV of the electron transport chain, cytochrome c oxidase, revealed that this population of mitochondria retains activity (Figure 1d, Figure S1). Complex IV activity was detected as a punctate staining pattern directly surrounding the plaque zone, mirroring the staining pattern for VDAC. Although it is currently unclear which cells are responsible for the plaque‐associated ring‐like clusters of mitochondria, the close proximity and staining pattern suggest that dystrophic neurites rather than glial populations are likely the source of mitochondrial enrichment. These data show that a distinct population of mitochondria accumulate near amyloid plaques, and that this phenomenon is conserved from mice, to monkeys, to humans.

### 2.2 Amyloid plaques autofluorescence and are heterogeneous in primates

To evaluate metabolism from a broader perspective, multiphoton metabolite‐based imaging was used to investigate the local impact of amyloid plaques. Nicotinamide adenine dinucleotide (NAD) and nicotinamide adenine dinucleotide phosphate (NADP) are central co‐factors in redox metabolism that autofluorescence in their reduced forms. Multiphoton laser scanning microscopy (MPLSM) imaging can be used to quantify the fluorescence intensity and fluorescence lifetime of NAD(P)H in cells and in tissue sections (Martin et al., 2016, 2018). Using near‐infrared excitation, MPLSM non‐invasively detects NAD(P)H autofluorescence and fluorescence lifetime imaging microscopy (FLIM) measures the latency between excitation and photon release. These quantitative measures reflect NAD(P)H levels and the cellular environment of NAD(P)H, respectively. Mean fluorescence lifetime (*τ*_m_) describes a first‐order decay curve including a fast component (*τ*_1_) and a slow component (*τ*_2_) that correspond to free and protein‐bound pools of NAD(P)H.

**FIGURE 2:**
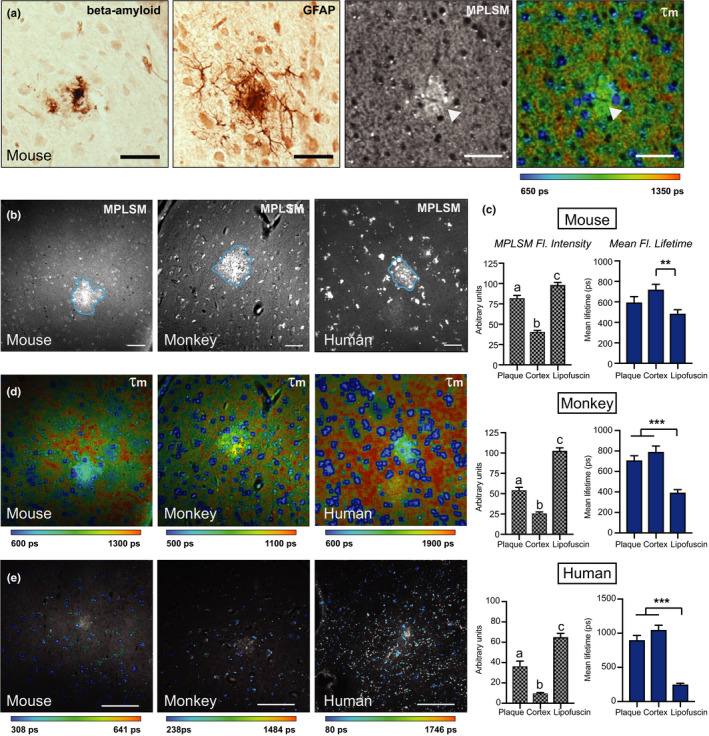
Multiphoton imaging identifies plaques and allows quantitation of lipofuscin. (a) Adjacent cryosections of APP/PSEN1 mouse cortex immunostained for beta‐amyloid, GFAP, and two‐photon fluorescence intensity (ex. 740 nm) and mean fluorescence lifetime (*τ*_m_) images of an unstained cortical section (20×). White arrows indicate plaque autofluorescence. (b) Representative two‐photon fluorescence intensity images of APP/PSEN1 mouse, monkey, and human AD cortical tissue (20×). Scale bar = 20 µm. (c) Quantification of two‐photon fluorescence intensity (gray) and mean lifetime (blue) of plaques, tissue, and lipofuscin particles between species. (d) Representative mean lifetime images of APP/PSEN1 mouse, monkey, and human AD cortical tissue. (e) Mean lifetime images of mouse, monkey, and human AD cortical tissue spectrally sorted for lipofuscin particles. Scale bar = 100 µm. Mouse (*n* = 5–6); monkey (*n* = 5); human (*n* = 10) biological with 6–18 plaque ROIs quantified within species; data are shown as average ± SEM; a‐c *p* < 0.001, **p < 0.01, ***p < 0.001, as determined by one‐way ANOVA with post hoc testing of differences between groups

In mice, MPLSM imaging identified bright autofluorescent foci. To investigate whether the plaques themselves might be responsible for the autofluorescence signal, three adjacent APP/PSEN1 brain sections were respectively stained using antibodies against glial fibrillary acidic protein (GFAP—a marker of astrocytes that increases upon astrocyte activation), antibodies against beta‐amyloid, or used unstained for MPLSM and FLIM. The autofluorescence aligned with immunodetection of the plaques (Figure 2a). Second harmonic generation (SHG) imaging is a multiphoton compatible method for detecting fibrillary collagen, including that seen in fibrosis (Keikhosravi et al., 2014). SHG did not detect the plaques as features, indicating that fibrillary collagen did not contribute to the autofluorescence signal. This was confirmed by histological detection of fibrosis using picrosirius red that picked up fewer than 10% of the plaques in monkeys (Figure S2). Autofluorescence of the amyloid plaque was also detected in monkeys and humans (Figure 2b). The fluorescence intensity was quantified by region of interest including the region of the plaque (~600 µm^2^) and the area encompassing 60 µm from the plaque edge. In all three species, the intensity of NAD(P)H detected was greater within the region of the plaque compared to the surrounding tissue (Figure 2c).

### 2.3 Lipofuscin is increased in aged monkeys and humans

Lipofuscin is a deposit of oxidized lipids, proteins, and metal ions that increases with aging and is known to autofluorescence (Moreno‐Garcia et al., 2018). Lipofuscin was readily detected by MPLSM, and could be spectrally isolated and quantified by FLIM due to the very short fluorescence lifetime (*τ*_m_) of lipofuscin‐associated photon decay (Figure 2c‐e). The total numbers of cortical lipofuscin particles were similar among species (Figure S2), but particle size was significantly greater in brains from monkeys than brains from mice, and greater still in brains from human subjects (Figure S2). Detection of large lipofuscin particle sizes was unique to monkeys and humans consistent with prior studies indicating that lipofuscin accumulation is age‐dependent (Benavides et al., 2002). The data presented here show that levels of lipofuscin are elevated in monkeys with amyloid burden and in human AD, and that aged monkeys recapitulate the human phenotype better than mice.

### 2.4 Greater plaque‐associated astrocyte activation in aged monkeys and humans

**FIGURE 3:**
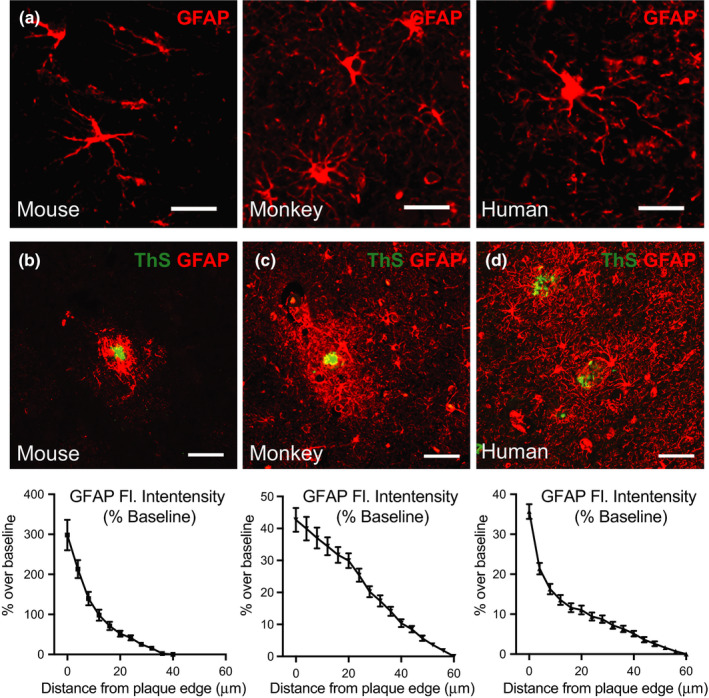
Astrogliosis in response to beta‐amyloid shows an age‐component. (a) Immunofluorescent labeling of astrocytes (GFAP) distal to amyloid plaques in APP/PSEN1 mouse, monkey, and human AD cortex (40×). Scale bar = 10 µm. (b‐d) Immunofluorescent labeling of astrocytes and amyloid plaques (Thioflavin S) in cortical tissue of (b) mice (c) monkeys (d) and AD cortex (20×), with quantification of fluorescent stain intensity relative to baseline. Scale bar = 40 µm. Mouse (*n* = 6); monkey (*n* = 5); human (*n* = 10) biological replicates with 39–210 plaque ROIs quantified within species; data are shown as average ± SEM

Astrocytes are critical in maintaining many aspects of neuronal homeostasis, including neurotransmitter recycling, nutrient delivery, and structural support (Kimelberg & Nedergaard, 2010). Consistent with prior reports (Vasile et al., 2017), cortical astrocytes appear larger with more numerous processes in monkeys and humans compared to mice (Figure 3a). In mice, astrocytes adjacent to neuritic plaques have been shown to become hypertrophic and inflammatory and participate in cross talk with microglia and neuronal populations (Lian et al., 2016). Astrocyte reactivity to beta‐amyloid has been documented in numerous animal models of AD (Rodriguez‐Arellano et al., 2016). Here, astrocyte reactivity was quantified by immunofluorescent detection of GFAP, with reference staining of amyloid plaques conducted in parallel using thioflavin S (ThS). In cortical sections from APP/PSEN1 mice, activated astrocytes were clustered in the immediate area surrounding amyloid plaques and resolved to baseline at ~36 µm from the edge of the plaque (Figure 3b, Table S2). GFAP abundance was also elevated in the amyloid plaque vicinity in monkeys (Figure 3c); however, the extent of astrocyte activation was significantly greater in monkeys than in mice, failing to resolve by 60 µm from the plaque edge (Table S2). In humans, astrocyte reactivity was also detected in the vicinity of amyloid plaques (Figure 3d), but similar to monkeys the activation zone extended to ~56 µm from the plaque edge. Greater variability in plaque‐associated astrocyte activation was observed in humans and in monkeys, in addition to considerable heterogeneity in baseline cortical GFAP expression (Figure S3). Moreover, in several instances, astrocytes within regions of high plaque density in humans displayed attenuated activation indicating that plaque density influences the ability of astrocytes to respond (Figure S3). For humans and nonhuman primates, heterogeneity in the impact of plaques was observed both among individuals and at the level of individual plaques within individuals (Figure S4). These data demonstrate that astrocyte number, structure, and zone of reactivity in equivalent in nonhuman primates and humans, and that age appears to be major factor contributing to astrogliosis that is not captured by the mouse model.

### 2.5 Extensive microglial recruitment in monkeys with amyloid burden

**FIGURE 4:**
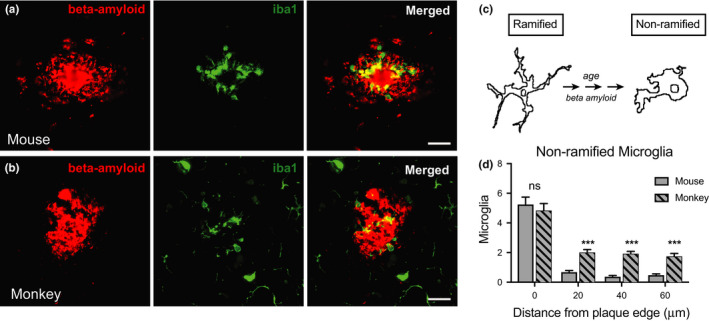
Increased basal and plaque‐associated microglia activation in the aged primate brain. Immunofluorescent labeling of microglia (iba1) and beta‐amyloid in cortical tissue of (a) APP/PSEN1 mice (b) monkeys (40×). Scale bar = 20 µm. (c) Schematic of morphological changes in ramified vs. non‐ramified microglia. (d) Numbers of non‐ramified microglia in 20 µm stratifications from the edge of amyloid plaques. Mouse (*n* = 6); monkey (*n* = 5) biological replicates with 55–60 plaque ROIs quantified within species; data are shown as an average ± SEM; ***p < 0.001, Multiple *t* tests

Microglia are brain‐resident macrophages that play diverse roles in clearing debris, pruning synapses, and mediating immune responses (Butovsky & Weiner, 2018). Beta‐amyloid is known to trigger microglial activation whereby microglia become hypertrophic and phagocytic (Colonna & Butovsky, 2017). Activation status can be inferred from microglial morphology. Activated microglia are amoeboid with large somas and attenuated process length in contrast to poised microglia that are ramified in shape with long branched processes and a small cell body. For this study, the status of microglia was evaluated using double immunofluorescent detection of beta‐amyloid and the microglial marker Iba1. Although similar numbers of total microglia were detected in the cortical sections from mice and monkeys, microglial morphology was strikingly different between species. Monkeys displayed more heterogeneity in microglial structure, including polynucleated somas and more heterogeneity in cell size, matching reports of human microglial diversity (Streit et al., 2020). In both mice and monkeys, enrichment of non‐ramified microglia was identified in the immediate vicinity of amyloid plaques. In mice, the activated microglial were limited to a small area directly surrounding the plaque (Figure 4a). In monkeys, a greater zone of microglial activation was detected (Figure 4b), and variability in microglial morphology was evident surrounding individual plaques and between plaques of the same individual (Figure S5). These data are consistent with both amyloid‐dependent microglial activation and microglial “priming” as a function of age (Figure 4c). Moreover, a greater number of non‐ramified microglia were observed in monkeys distant from plaques, suggesting a degree of age‐related but amyloid‐independent microglial activation (Figure 4d). Collectively, these data demonstrate that there are differences in microglial structure and activation state between species, and highlight differences in cellular composition within the amyloid plaque microenvironment between mice and monkeys.

## 3 DISCUSSION

Challenges remain in Alzheimer's disease research that are likely to be overcome with the new injection of funding and increased research activity in this area. In terms of modeling the disease, there are some limitations in the most common approaches. Mice do not naturally develop plaques and tangles and so the etiology of the early stages of the disease cannot be readily explored in current models. Most transgenic mouse models used to date are better reflection of early‐onset AD rather than late‐onset AD and do not consider age as a contributing factor. Several aspects of brain aging are unique within species, such as intrinsic differences in glial biology, and those important to AD onset and progression may be primate‐specific. The data in this study suggest that amyloid accumulation in an aged environment differentially impacts inflammatory processes that we know are linked to neurodegeneration. We report differences in cellular composition, morphology, and metabolism in the immediate vicinity of amyloid plaques. Some features are shared among the three species under investigation but others appear only in the primate models and may explain why common mouse AD models do not recapitulate the functional deficits associated with human disease. In this study, we show striking parallels between the aged monkey model and late‐onset human disease in the localized impact of amyloid, arguing that studies in aged nonhuman primates may provide a means to advance our understanding of the biology and etiology of AD. The data presented here are consistent with a role for mitochondria in the amyloid plaque environment. Mitochondria were enriched in the immediate vicinity of amyloid plaques in APP/PSEN1 mice, monkeys, and human AD cortex. Elevated cytochrome c oxidase activity was detected in this population of mitochondria in mice, suggesting that they are functionally different; however, the nature of this distinction and its physiological consequence remain to be determined. Mitochondrial adaptation could represent a compensatory response in dystrophic neurites to amyloid toxicity. Conversely, the clustered mitochondria could be maladaptive and contribute to elevated oxidative stress and metabolic insufficiency. The phenotype we describe is distinct from prior reports showing that mitochondrial dysfunction is a major feature of AD pathology. The “mitochondrial cascade hypothesis” has been proposed, which suggests that bioenergetic deficiency mediates AD neurotoxicity (Swerdlow et al., 2014). The biology behind this clustering of mitochondria in the immediate area of the plaque has yet to be defined, but the fact that it appears to be conserved among species argues to its importance in the response to AD pathology.

A unique fluorescent signature was detected by MPLSM that appears to co‐localize with the clustered mitochondria and the amyloid plaques themselves. This signature was conserved for all three species and is characterized by higher fluorescence intensity and lower mean fluorescence lifetime compared to surrounding tissue. Data shown here suggest that fibrillary beta‐amyloid at least partially responsible for this autofluorescence; however, mitochondrial NADH also produces strong fluorescence at near‐infrared excitation. The use of a genetically encoded NADH/NAD^+^ biosensor with cellular resolution has been reported in brain slices (Mongeon et al., 2016); such a probe would be useful in differentiating between plaque fluorescence and NADH in situ. Intraneuronal lipofuscin is striking feature of age that was captured using MPLSM and FLIM in our study. Lipofuscin has been reported to increase with age in humans where it is speculated to represent dysfunctional autophagy in aged neurons (Benavides et al., 2002; Moreira et al., 2010). Accumulation of lipofuscin has been implicated in the pathology of neurodegenerative diseases, and a role for lipofuscin in AD has been proposed (Giaccone et al., 2011). In this study, intraneuronal deposits of lipofuscin were observed in monkey and human cortex, and were much smaller than those in young APP/PSEN1 mice. The short fluorescence lifetime of lipofuscin particles was conserved among species, suggesting perhaps some overlap in the composition of these deposits; however, differing biochemical properties of lipofuscins have been reported in cortical tissue over the course of aging (Benavides et al., 2002).

The activation of astrocytes and microglia surrounding neuritic plaques has been documented in animal models and postmortem AD brain (De Strooper & Karran, 2016). The data presented here show that astrocytes in the aged monkey brain are more numerous, larger in size, and display greater number of processes than their young mouse counterparts. It is possible that late‐induction of AD pathology in an already aged mouse could produce this same effect but this model is not currently available. We would draw a distinction between a constitutive transgenic AD mouse that is aged out and the induction of AD pathology in a mouse that is already aged. The former would inform about the reactivity to life‐long pathology but the latter would inform of the contribution of the aged brain environment in the response to emerging AD pathology. Plaque‐associated astrocyte reactivity impacted a greater area in monkeys and humans than in mice, but here too, it is unclear whether these differences are the result of differences in age or due to differences among species. A further consideration is that AD pathology also includes neurofibrillary tangles that have not been considered in this study. Both humans and monkeys develop tau pathology as a function of age, and it is not always accompanied by amyloid deposition (Arnsten et al., 2021; Paspalas et al., 2018). It is possible that some of the shared features identified in monkeys and humans could be related to this age‐related phenotype that is not modeled in the APP/PSEN mouse.

Astrocytes are more densely populated in the primate cortex (Vasile et al., 2017), being larger in size and more sophisticated in morphology. Furthermore, astrocytes in the aged brain could be more sensitized to activation. Accumulating evidence points to a central role for microglia in AD risk and severity (Colonna & Butovsky, 2017). In this study, microglial morphology and distribution was uniform around each plaque and limited to the immediate plaque vicinity in mice, a finding that is consistent with previous studies suggesting that this constitutes an acute‐phase response to a protective barrier, limiting plaque expansion and neurotoxicity (Condello et al., 2015). The same was not true in monkeys, where microglia surrounding plaques adopted a spectrum of morphologies, were often polynucleated, and were variable in size and ramification. The morphological diversity of the amyloid‐proximal microglial population in monkeys may correspond to subpopulations that differ in activation state. Alternatively, the heterogeneity of microglia in the monkey brain could represent dystrophy of the plaque‐associated population. Similar abnormalities in microglial structure have been reported in postmortem AD brains (Streit et al., 2020). In monkeys, amoeboid microglia were also observed in stratifications distal to plaques, suggesting amyloid‐independent microglial priming in monkeys as a function of age, although species‐dependent differences cannot be ruled out. Data shown here demonstrate that the trajectories of amyloidosis and glial reactivity differ among individual monkeys and humans, reflecting the complexity of the disease and the important role that brain aging has on disease pathogenesis. The development of a mouse model with a later emergence of AD pathology could be more physiologically relevant to late‐onset AD; however, this strategy would not overcome potential species differences in glial status and reactivity. Given the clear parallels in the local impact of amyloid plaques between monkeys and humans, we suggest studies devised using nonhuman primate models would are likely to translate to human disease and that interventions developed from these studies would have significant potential for clinical efficacy.

## 4 EXPERIMENTAL PROCEDURES

### 4.1 Animal models

#### 4.1.1 Mice

Male C57BL/6J (APP/PSEN; mice (*n* = 6) were obtained from Jackson Laboratories at 4 months of age (strain ID: B6. Cg Tg (APPswe, PSEN1dE9) 85Dbo/Mmjax; Stock #34832; Bar Harbor, ME, USA) and housed under controlled pathogen‐free conditions in accordance with the recommendations of the University of Wisconsin Institutional Animal Care and Use Committee. Mice were fed 87 kcal week of control diet (F05312, Bio‐Serv, Flemington, NJ, USA) and were individually housed with ad libitum access to water. Mice were euthanized at 6 months of age. Brains were isolated, bisected, embedded in OCT, frozen in liquid nitrogen, and stored at −80°C until further processing.

#### 4.1.2 Rhesus macaques

Male and female rhesus monkeys (*n* = 5) between 29 and 35 years of age (average age = 31.1 years at necropsy) were included in this study. The animals were part of the Aging and Caloric Restriction longitudinal study conducted at the Wisconsin National Primate Research Center. Details of housing and husbandry have been previously described (Colman et al., 2009; Mattison et al., 2017; Ramsey et al., 2000). Animals were fed a semi‐purified diet (13% protein; 60% carbohydrate; 11% fat) that limited year‐to‐year and seasonal variation in nutrient sources. Upon euthanasia, brains were harvested, sectioned according to a standard necropsy protocol, fixed overnight in 10% neutral‐buffered formalin, and paraffin‐embedded.

### 4.2 Human tissue

Brain tissue from male and female Alzheimer's disease subjects (*n* = 10) between 72 and 96 years of age (average age = 86.5 years at necropsy) were included in this study. Tissue was acquired from the Wisconsin Alzheimer's Disease Research Center (ADRC) brain bank, and comprised cases from the ADRC clinical core. All cases had a postmortem diagnosis of AD following the National Institute of Aging and the Alzheimer Association criteria for neuropathological AD (Hyman et al., 2012). Frozen tissue was sectioned and fixed in 10% neutral‐buffered formalin prior to histological analysis.

### 4.3 Histochemistry and immunodetection

For APP/PSEN1 mouse and AD cortical tissue, serial cryostat sections 10 μm in thickness were cut at −15°C with a Leica Cryostat (Fisher Supply, Waltham, MA, USA), defrosted and air dried, and used for histological staining. For rhesus cortical tissue, serial microtome sections 5 μm in thickness were cut with a Leica Microtome (Fisher Supply) and sections were deparaffinized prior to histological staining. Cytochrome c oxidase activity staining was performed on mouse sections as previously described (Pugh et al., 2013). For immunodetection, sections were defrosted and air dried, fixed for 15 min in 10% neutral‐buffered formalin, permeabilized, and blocked for 30 min in Superblock buffer containing 0.3% Triton‐X. Tissue sections were incubated with primary antibodies overnight at 4°C in a humidified slide chamber. Thioflavin S staining was performed subsequent to immunostaining by incubating slides in 0.02% aqueous Thioflavin S solution, followed by clearing in 50% ethanol for 5 min. Antibodies and reagents used are as follows: AlexaFluor 488 anti‐rabbit or mouse IgG, AlexaFluor 594 anti‐rabbit or mouse IgG (Invitrogen), biotinylated anti‐mouse or rabbit IgG (Vector Labs, Burlingame, CA, USA), peroxidase‐labeled avidin–biotin complex (ABC) solution (Vector labs), ImmPACT NovaRED reagent (Vector Labs), GFAP (ab7260; Abcam), Iba1 (019‐19741; Wako), VDAC (ab154856; Abcam), beta‐amyloid (NBP2‐13075; Novus Biologicals), and Thioflavin S (T1892; Sigma‐Aldrich).

### 4.4 Image capture and analysis

Slides were imaged with a Leica (Buffalo Grove, IL, USA) DM4000B microscope and photographed with a Retiga 4000R digital camera (QImaging Systems, Surrey, BC, Canada). Camera settings were optimized for each stain; for uniformity, all images for a given stain were taken on the same day with identical settings, fixed light levels, and fixed shutter speed optimized at each magnification. Digital images were converted to grayscale prior to image analysis, and image analysis was performed using FIJI software. Analysis of GFAP and VDAC fluorescence in the vicinity of plaques was performed as follows: Plaque ROIs were manually created based on Thioflavin S or beta‐amyloid immunofluorescence. Stratifications of set distances from the edge of the plaque were defined out to 40 μm or 60 μm using the ROI manager. Prior to stain quantification, a uniform intensity threshold was applied to eliminate fluorescence equivalent to unstained areas of the slide. The same threshold was applied to all images from the same imaging session. To preserve differences in fluorescence intensity within each image, no background subtraction was applied. Baseline fluorescence intensity of each target was defined by the fluorescence intensity at 60 μm from the edge of each plaque. Plaques with overlapping ROIs were included in the analysis. For analysis of microglia, plaque ROIs were generated using anti‐beta‐amyloid immunostaining. Three stratifications of 20 μm intervals were defined and microglia within each stratification were manually counted. Non‐ramified microglia were identified by soma enlargement and retraction of processes.

### 4.5 Multiphoton laser scanning microscopy

One day prior to multiphoton imaging, cryostat or deparaffinized sections were dried onto glass coverslips and mounted using Cytoseal 60 mounting solution (Richard Allen). The instrument response function of the optical system was calibrated before each imaging session, and sections were imaged at 20× magnification. Data were collected using an excitation wavelength of 740 nm, and emission was filtered at 457 ± 50 nm, the spectral peak for NADH/NADPH. The data collection time was 240 s using a pixel frame size of 256 × 256. The system has a H7422P‐40 GaAsP photon counting PMT (Hamamatsu) for intensity and lifetime imaging. Acquisition was performed with WiscScan, an acquisition software package developed by the UW‐Madison Laboratory for Optical and Computational Instrumentation. Autofluorescence intensity and fluorescence lifetime data were analyzed in SPCImage (Becker & Hickl, v.3.9.7, Berlin, Germany) where a Levenberg–Marquardt routine for nonlinear fitting was used to fit the fluorescence decay curve collected for each pixel in the 256 × 256 frame to a model multi‐exponential decay function. Data were assessed by the minimized chi‐square value generated during the fit so that analysis was unbiased. To eliminate background fluorescence a threshold for analysis was applied based on photon counts. Additionally, pixels were assigned a bin of 3 for optimal fitting of the data. Autofluorescence intensity data were analyzed using FIJI (NIH, Wayne Rasband; https://imagej.nih.gov/ij/). ROIs for amyloid plaques lipofuscin particles were defined based on autofluorescence intensity and used for subsequent analysis.

### 4.6 Statistical analysis

Mean stain intensity for GFAP, VDAC, and cytochrome c oxidase activity assays was quantified in each stratification from the plaque and normalized to “baseline” stain intensity at 40 μm or 60 μm from the plaque edge. Statistical analysis for differences in GFAP and VDAC stain intensity from baseline was performed using linear mixed models (LMM) to account for the dependency among plaque measurements per animal. The minimum variance quadratic unbiased estimator (MIVQUE0) approach to LMM was used to determine differences in means between stratifications. For quantification of activated microglia, individual Student's *t* tests were performed on each stratification from the edge of plaques using pooled averages of samples within each species. Lipofuscin particles detected using MPLSM were defined based on autofluorescence intensity and quantified in FIJI. Mean values for lipofuscin particle size and number were averaged between samples within each species and evaluated using an ANOVA with post hoc analyses.

## CONFLICT OF INTEREST

The authors declare no conflict of interest.

## AUTHOR CONTRIBUTIONS

The study was designed by DCS and RMA. DCS, IAD, ABS, SW, and SAM conducted experiments, generated, and analyzed data. AKS and KWE consulted on the multiphoton imaging (SHG and FLIM). SMS and BBB assisted with human specimen acquisition. SMS was involved in the neuropathology and diagnosis of the human specimens. RJC assisted with rhesus monkey specimen acquisition. TMB provided statistical expertise. DCS and RMA wrote the paper.

## Supporting information

Supplementary Material

## Data Availability

All data and protocols will be made available upon request.
