# Supplementary material for: Rhesus monkeys as a translational model for late‐onset Alzheimer's disease
Source: Aging Cell. 2021 May 5;20(6):e13374. doi: 10.1111/acel.13374 (PMC8208787; doi:10.1111/acel.13374)

Fig.S1: Mitochondria accumulate around amyloid plaques. (a) Box and Whisker plots of median plaque size, IQR, and range between species. (b) Scatter plots of individual plaque sizes between species. (c) Immunofluorescent labeling of mitochondria (VDAC) and amyloid plaques (Thioflavin S) in human AD cortex. Scale bar = 20 μm. (d) Immunofluorescent labeling of mitochondria in human AD cortex with orange lipofuscin deposits (yellow arrow) and red mitochondria (white arrow). (e) Adjacent sections of monkey cortex stained for beta amyloid and VDAC with cryosection of human AD cortex stained for VDAC. Red arrow corresponds to plaque-associated mitochondria. Scale bar = 50 μm. (f) Quantification of cytochrome c oxidase activity in 4 μm increments from the edge of amyloid plaques in APP/PSEN1 mice. Mouse (n=5-6); Monkey (n=5); Human (n=10) biological replicates with [n=39-294 plaque ROIs quantified within species]; data are shown as an average ± SEM; **$p < 0.01$, ***$p < 0.001$, One-way ANOVA; For cytochrome c oxidase activity, *$p < 0.05$, linear mixed model (MIVQUE0).

Fig.S2: Multiphoton imaging identifies plaques and allows quantitation of lipofuscin. (a) Adjacent sections of monkey cortex immunostained for beta amyloid and stained with picrosirius red for collagen (20x). Black arrow indicates plaque-associated fibrosis. Scale bar = 50 μm. (b) Quantification of lipofuscin particle number between species. (c) Quantification of lipofuscin particle size between species. Mouse (n=5); Monkey (n=3); Human (n=4-5) biological replicates with [n=3-15 plaque ROIs quantified within species]; data are shown as an average ± SEM; a-c $p < 0.001$, One-way ANOVA with post-hoc analysis.

Fig.S3: Astrogliosis in response to beta-amyloid shows an age-component. (a) Immunofluorescent labeling of astrocytes (GFAP) in mouse, monkey, and human AD cortex with 3 biological replicates within groups (20x). (b) Immunofluorescent labeling of astrocytes and amyloid plaques (ThS) in human AD tissue (20x). Scale bar = 40 μm.

Fig.S4: Astrogliosis is heterogenous between individuals and plaques. (a) Heatmap of plaque associated GFAP immunoreactivity as a percentage of baseline between individual samples. Blue stars indicate unresolved astrogliosis. (b) Heatmap of plaque associated GFAP immunoreactivity as raw values between plaques.

Fig.S5: Microglial morphology is heterogenous in monkeys (a) Immunofluorescent labeling of plaque-associated microglia (iba1) in monkeys showing activated microglia (white arrow) and dystrophic microglia (yellow arrow). Scale bar = 30 μm.

Sample	Sex (M/F)	Age	Sample Region
APP/PSEN1 Mice	M	6 mo	somatosensory cortex
Stock No: 34832-JAX	M		
(n=6)	M		
	M		
	M		
	M		
Rhesus Macaque	M	30.4	temporal cortex
(n=5)	M	30	
	M	31.1	
	M	29.3	
	F	34.8	
Human AD	M	72	middle temporal gyrus
(n=10)	M	82	post. middle temporal gyrus
	M	84	middle temporal gyrus
	M	95	middle temporal gyrus
	M	96	middle temporal gyrus
	F	72	middle temporal gyrus
	F	86	middle temporal gyrus
	F	87	post. middle temporal gyrus
	F	95	post. middle temporal gyrus
	F	96	post. middle temporal gyrus

Table S1: Characteristics and sample region of animal and human subjects

Measure	Mouse	Monkey	Human
VDAC	28µm	No Resolution	NA
Cyt c ox.	28µm	NA	NA
GFAP	36µm	No Resolution	56µm

Resolution of signal to baseline; $p < 0.05$, linear mixed model (MIVQUE0)

Table S2: Resolution of amyloid plaque-associated immunostaining to baseline

Figure S1

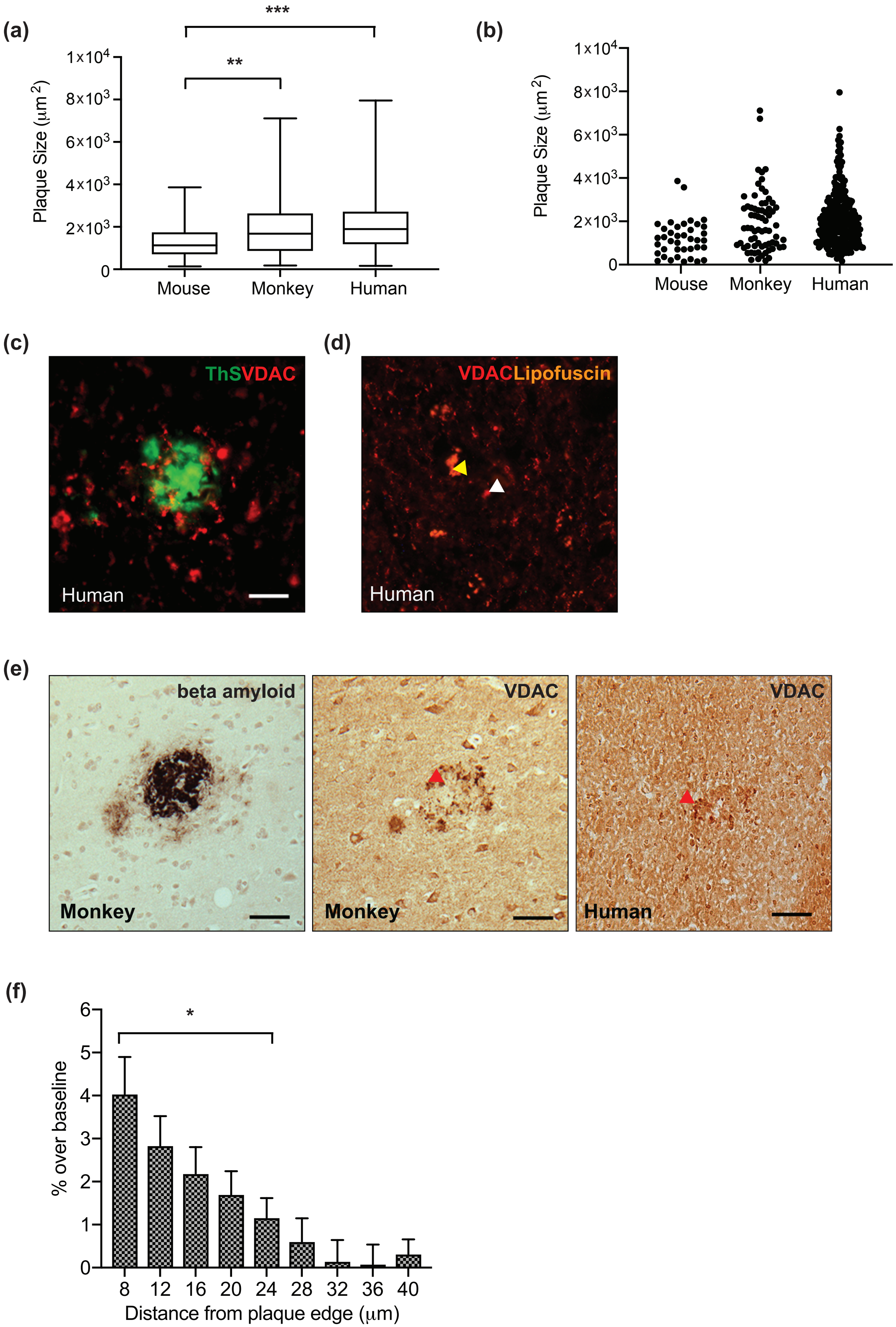
(a)
***
**
Plaque Size (μm2)
1x104
8x103
6x103
4x103
2x103
0
Mouse
Monkey
Human
(b)
Plaque Size (μm2)
1x104
8x103
6x103
4x103
2x103
0
Mouse
Monkey
Human
(c)
ThSVDAC
Human
(d)
VDACLipofuscin
Human
(e)
beta amyloid
Monkey
VDAC
Monkey
VDAC
Human
(f)
*
% over baseline
6
5
4
3
2
1
0
8
12
16
20
24
28
32
36
40
Distance from plaque edge (μm)

Figure S2

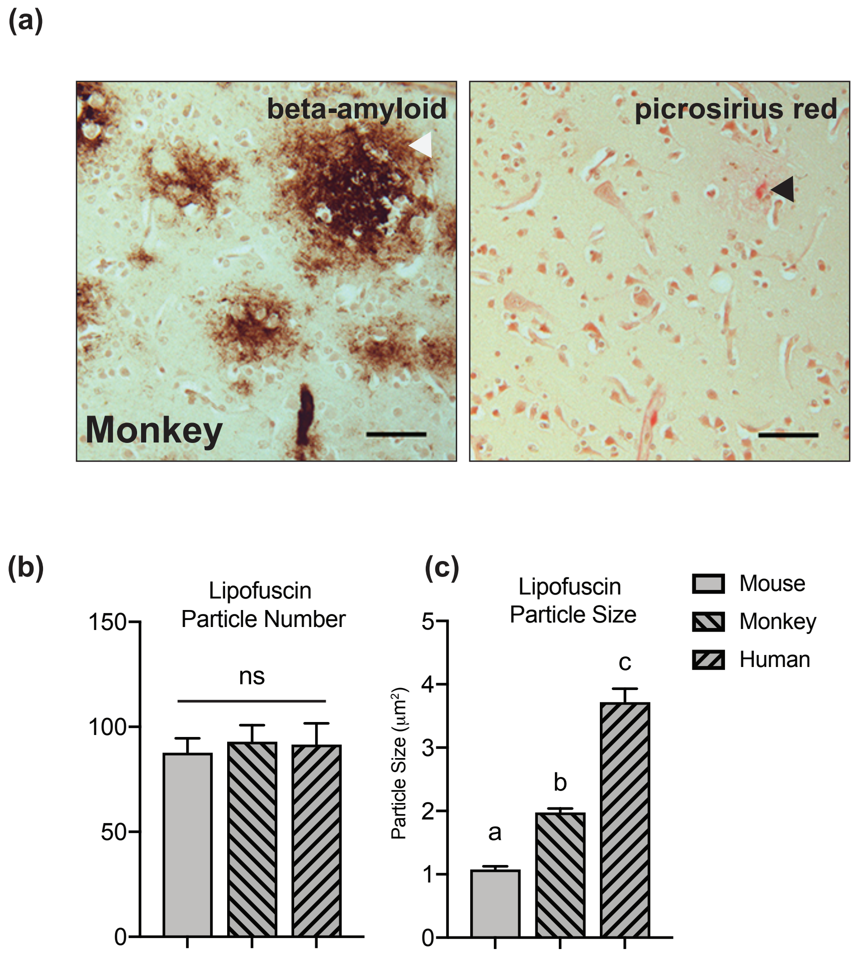

(a)
beta-amyloid
Monkey
picrosirius red
(b)
Lipofuscin
Particle Number
150
100
50
0
ns
(c)
Lipofuscin
Particle Size
Particle Size (μm²)
5
4
3
2
1
0
a
b
c
Mouse
Monkey
Human

Figure S3

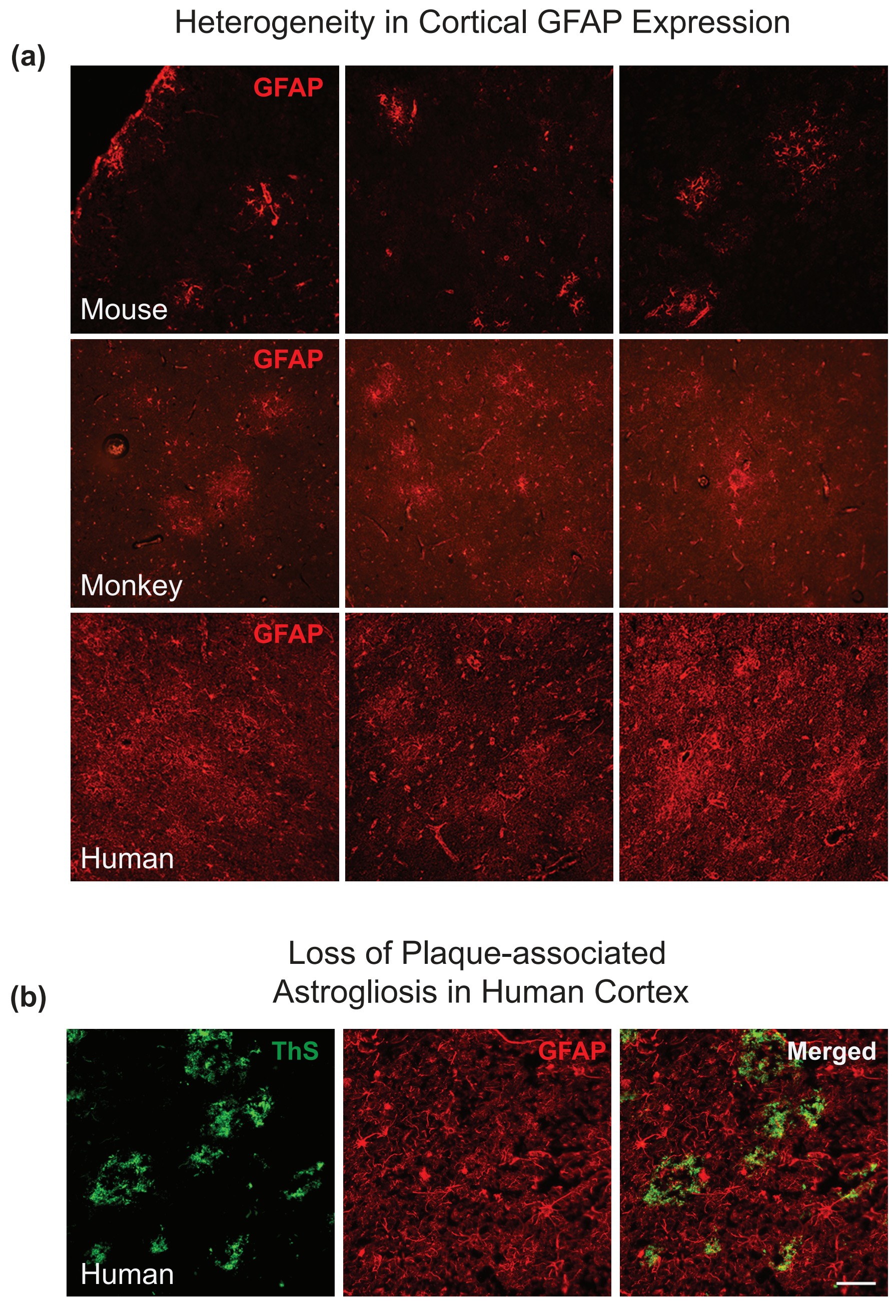
Heterogeneity in Cortical GFAP Expression
(a)
GFAP
Mouse
GFAP
Monkey
GFAP
Human
Loss of Plaque-associated
Astrogliosis in Human Cortex
(b)
ThS
Human
GFAP
Merged

Figure S4

(a)

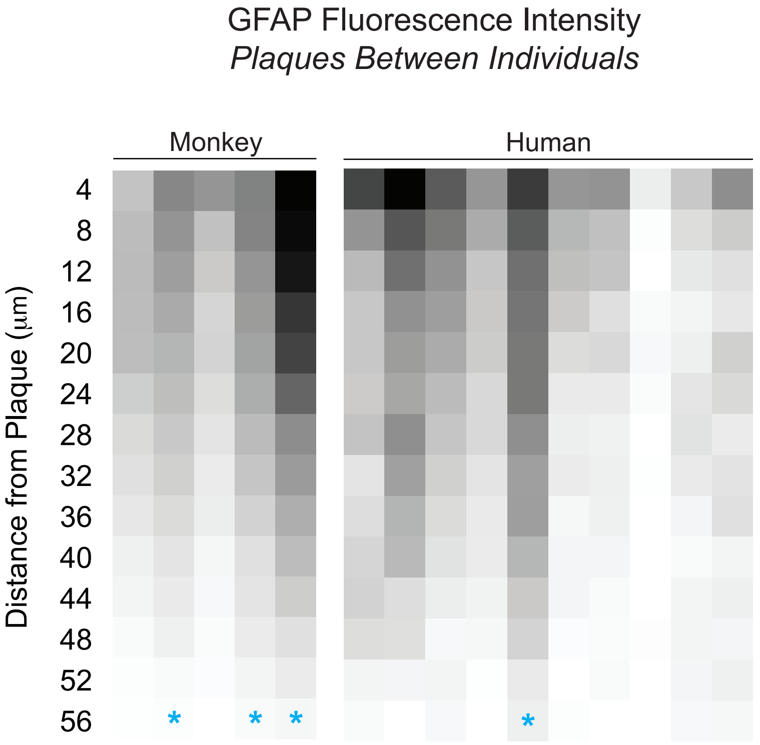
GFAP Fluorescence Intensity
Plaques Between Individuals
Monkey
Human
Distance from Plaque (μm)
4
8
12
16
20
24
28
32
36
40
44
48
52
56
*

(b)

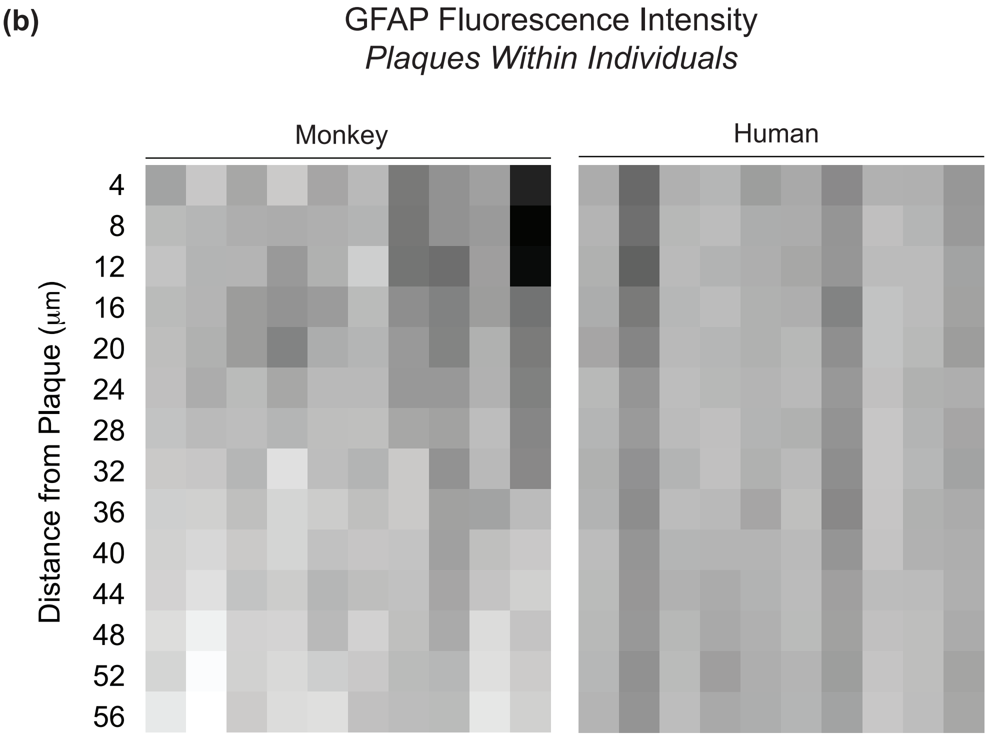
GFAP Fluorescence Intensity
Plaques Within Individuals
Monkey
Human
Distance from Plaque (μm)
4
8
12
16
20
24
28
32
36
40
44
48
52
56

Figure S5

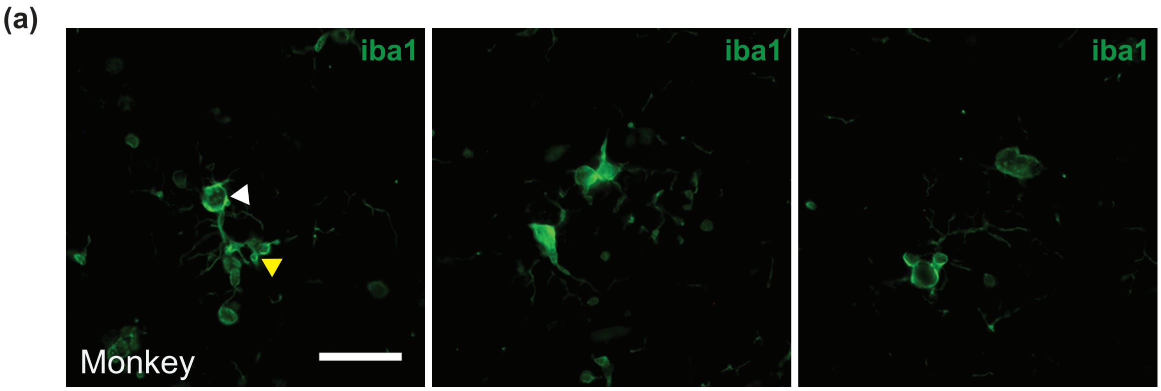

Supplement: Supplementary file 1 — Supplementary Material [file ACEL-20-e13374-s001.pdf]
